# Sequestration and the Extended Museum Specimen: Effects of Time and Preparation Methodology

**DOI:** 10.1007/s10886-025-01646-7

**Published:** 2025-09-29

**Authors:** Megan E. Zabinski, M. Deane Bowers

**Affiliations:** 1 Department of Ecology and Evolutionary Biology, University of Colorado, Boulder, CO USA; 2 Department of Ecology and Evolutionary Biology and Museum of Natural History, University of Colorado, Boulder, CO USA

**Keywords:** Iridoid glycosides, Sequestration, Museum collections, Extended museum specimen

## Abstract

Billions of specimens are housed in museum collections, but only a fraction are used in research after accession. The chemical composition of museum specimens, such as the presence of plant secondary metabolites (PSMs), is a useful component of the extended museum specimen concept. However, most of the work investigating chemistry of museum specimens has focused on herbarium specimens and not insects, of which some species may sequester and retain plant secondary metabolites. Also, the preparation techniques involved in entomological collections vary substantially and may influence the ability to detect sequestered compounds. The present work uses two *Euphydryas* butterfly species, *E. phaeton* and *E. anicia* (Nymphalidae), that sequester one group of plant secondary metabolites, iridoid glycosides, to ask two questions about the detection of sequestered compounds in museum specimens: (1) Can sequestered iridoid glycosides be detected in insect museum specimens and if so, how far back in time can we detect them? and (2) Do the preparation techniques used on insect specimens impact sequestered iridoid glycoside content? We detected sequestered compounds in insect museum specimens dating back to 1933. We also found that freshly collected specimens had significantly higher amounts of stored defenses compared to all other treatments. This work highlights the potential for use of museum specimens in chemical ecology and informs collections interested in adding chemical content to their extended museum specimen data.

**Supplementary Information:**

The online version contains supplementary material available at 10.1007/s10886-025-01646-7.

## Introduction

There are billions of specimens housed in museum collections worldwide, but only a small fraction of these are used in research after accession, despite their documented utility (Shultz et al. 2021; Bakker et al. 2020). Museum specimens are used in many important ways, such as elucidating taxonomic and evolutionary relationships of both extant and extinct taxa (Burrell et al. 2015; Grewe et al. 2021), documenting migratory patterns (Freedman et al. 2020), and for examining the genetic consequences of climate change and pollution (DuBay and Fuldner 2017; Schmitt et al. 2019). However, more recently the “extended museum specimen”, which links other extractable data such as genetics, content of isotopes, dietary composition, and behavior to specimens has become an important focus of museum collections (Cook et al. 2016; Webster 2017; Lendemer et al. 2020). As technology advances and we can extract more data from already curated specimens, the utility of museum collections continues to grow.

The chemical composition of museum specimens, such as the presence of DDT in bird eggs (Ratcliffe 1967; Hickey and Anderson 1968) and the presence of secondary metabolites in plant specimens (e.g. Phillipson 1982) are examples of the extended museum specimen. The use of chemical analysis to identify plant secondary metabolites in herbarium specimens dates to the early 1950’s, with identification of alkaloids in certain plant species (Webb 1950; 1952). Since then, research on chemical composition of herbarium specimens has provided information on population structure (Berenbaum and Zangerl 1998; Moris et al. 2021), evolution of biosynthesis of chemical defenses (Mithen et al. 2010), and even the anthropological and pharmacological importance of plant chemicals (El-Seedi et al. 2005; Zangerl and Berenbaum 2005).

Much less common is the investigation of chemical compounds in insect museum specimens. These compounds may be produced *de novo* by insects or sequestered from the plants or prey on which they feed (Duffey 1980; Heckel 2014; Dettner 2015). One of the earliest examples of this research was the report of unidentified sequestered cardenolides reported from an adult museum specimen of the swallowtail butterfly, *Papilio antimachus* (Papilionidae), dated from 1900 (Table 1 in Rothschild 1972). More recently, cuticular hydrocarbons have been identified from museum wasp (Hymenoptera) species (Martin et al. 2009; Moris et al. 2021; Moore et al. 2021) and lucibufagins from museum firefly (Coleoptera) specimens (Berger et al. 2021). Although early discoveries of sequestered compounds in museum specimens have been documented (Rothschild 1972), this phenomenon has only been rarely investigated. If these compounds can be detected in historical specimens, identifying the timeline in which these specimens are viable for chemical analysis would be useful for identifying patterns of host plant use, history of predation pressure, and changes in other trophic interactions.

**Table 1:** Treatment abbreviations and full names; full name components represent preparation steps. Time in each step was standardized across treatments

Abbreviation	Full name
FE	Freeze – Extract
FAE	Freeze – Airdry – Extract
FOE	Freeze – Oven Dry – Extract
FSE	Freeze – Spread – Extract
FRSE	Freeze – Relax – Spread – Extract
KAE	Killjar – Airdry – Extract
KSE	Killjar – Spread – Extract
KRSE	Killjar – Relax – Spread – Extract

Investigation of sequestered defenses in insect museum specimens is rare (but see Nash et al. 1992; Berger et al. 2021; Petschenka et al. 2022). The ability to detect chemical compounds in museum specimens can be influenced by a variety of factors and two of the most important are time a specimen has been in the collection (how old it is) and specimen preparation methods, which have been well-studied for DNA (Dean and Ballard 2001). For example, DNA may degrade over time (Quicke et al. 1999), and the preservative used (e.g., formaldehyde versus ethanol) will influence the availability of DNA for sequencing (Post et al. 1993; Reiss et al. 1995; Koch et al. 1998). However, similar efforts have not been made to identify factors which could influence detection of sequestered or *de novo* produced compounds in museum insect specimens and, therefore, standardization of preparation and preservation is often lacking. For insects, collection and preservation techniques can differ in trapping, killing, and preparation methods that may vary by situation, collector, or institution. Often, not only does time spent in one of these steps vary, but the method and sequence of preparation techniques is rarely standardized or even documented for most museum specimens.

In the present study, we investigated the utility of insect museum specimens for application in chemical ecology on two fronts: the ability to detect sequestered chemical defenses in historic specimens and the effect of different preparation techniques on detection of these compounds in freshly collected specimens. This project uses two nymphalid butterfly species, *Euphydryas phaeton* and *Euphydryas anicia*, that sequester one group of plant secondary metabolites, iridoid glycosides (IGs), to answer two questions: *Question 1*: Can iridoid glycosides be detected in insect museum specimens and if so, how far back in time can we detect them? and *Question 2*: Do the preparation techniques used on insect specimen preparation impact iridoid glycoside content?

## Methods and Materials

### Study System

We used specimens housed in the University of Colorado’s Museum of Natural History (CUMNH) as well as freshly collected specimens of two *Euphydryas* species (Lepidoptera, Nymphalidae): *E. anicia* (collected in Boulder County, CO) and *E. phaeton* (laboratory reared from caterpillars collected in Burlington County, VT). Both species sequester the iridoid glycosides aucubin and catalpol as larvae and retain them to the adult stage, but *E. phaeton* typically sequester higher amounts of IGs (Stermitz et al. 1986; Bowers and Puttick 1986; Gardner and Stermitz 1988). Iridoid glycosides are polar compounds and are sequestered primarily in the hemolymph (Bowers 2003). Considered chemical specialists, these butterflies use IGs as both feeding and oviposition stimulants and larvae feed on a wide array of plants containing them (Stermitz et al. 1986; Ehrlich et al. 1975; Singer 1983; Bowers et al. 1992). These two species were chosen due to their sequestration ability, abundance in the CUMNH entomology collection, and ease of obtaining live *E. anicia* and *E. phaeton* adult specimens for comparisons.

We used historic museum specimens from both *Euphydryas* species (*anicia* and *phaeton*) to determine if IGs can be detected in museum specimens and if so, how old these specimens could be and still contain detectable amounts of these compounds. We compared percent dry weight of the total iridoid concentration (both aucubin and catalpol) to compare between species and among treatment groups. Using percent dry weight allows comparisons that are independent of variation in levels of hydration in individual specimens (Gilbert 2011). We calculated percent dry weight using the following formula: (milligrams of IGs/milligrams of dry specimen)*100. We compared IG content of these historic specimens with that of freshly collected specimens of both species (Fig. S1).

We also used *E. phaeton* butterflies reared in the laboratory to examine how preparation methods might affect IG content (Fig. S2). Female *Euphydryas* adults lay large egg clutches which include IGs (Bowers and Puttick 1986); therefore, to minimize between-sex IG variation, we used only males for both current and historic specimens. These butterflies were reared on *Chelone glabra*, a plant which contains primarily catalpol and smaller amounts of aucubin (Bowers et al. 1993).

*Question 1*: *Can iridoid glycosides be detected in insect museum specimens and if so*,* how far back in time can we detect them?*

Specimens of both *Euphydryas* species were obtained from the University of Colorado Boulder Museum of Natural History entomology collection (referred to as “historic specimens”) which contains specimens dating back to the early 20th century. To maintain representation in the collection, we used specimens from collecting bouts that contained multiple specimens (5+) that represent dates throughout a span of the 20th century. Six *E. phaeton* specimens were collected from 1936 to 1977 and nine *E. anicia* specimens were collected from 1933 to 1998 from various locations around the USA (Table S1). We first conducted a pilot experiment where we chemically extracted and analyzed only one set of wings (one forewing and one hindwing) from historic samples and compared them to IG quantities in the remaining specimen body. Unfortunately, sequestered iridoid glycosides were extremely low or undetectable in wing samples compared to remaining body samples, which led to the present experimental design of preserving one set of wings for the collection and using the rest of the specimen (body and left pair of wings) for chemical analysis in historic specimens. For each specimen, the right set of wings (forewing and hindwing) was removed and preserved in the collection, with the original data labels and barcodes. These historic specimens were then compared with freshly collected specimens reared or collected as described below.

*Question 2*: *Do the Preparation Techniques Used in Insect Specimen Preparation Impact Iridoid Glycoside Content?*

### Rearing of Fresh Specimens

Post-diapause *E. phaeton* larvae (4th and 5th instar) were collected from field sites in Burlington County, VT, USA during the late spring in 2021, brought back to Boulder, CO, and fed their native host plant *Chelone glabra* (Plantaginaceae) (also known as “white turtlehead”). These plants had been over-wintered outside and brought into the greenhouse at the University of Colorado Boulder in late April to allow regrowth. Caterpillars were initially placed in 10.16 cm x 15.24 cm x 7.62 cm clear plastic rectangular containers with mesh holes for air flow in groups of ~ 20–150 individuals per container in a growth chamber, which was maintained on 14:10 L: D cycles, at 25 °C light and 20 °C dark. These containers were lined with a paper towel, misted, and cleaned daily. Adults emerged and were allowed to mate; females oviposited egg clutches on *C. glabra*. Pre-diapause larvae were reared in groups on *C. glabra* until entering diapause in August. After entering diapause, larval groups were overwintered in a refrigerator at ~ 4 °C, misted and cleaned weekly to minimize disruption to overwintering larvae through physical movement as well as light and temperature changes. In late April, overwintering caterpillars were taken out of the refrigerator and allowed to sit at ambient room temperature (~ 22 °C) for ~ 2 weeks; as larvae began to come out of diapause, dead larvae were removed. At this stage, larvae had access to small amounts of host plant (*C. glabra*) in their containers to ensure access to food for individuals emerging from diapause. Surviving post-diapausing larvae were placed back into the growth chambers and maintained on 14:10 L: D cycles, at 25 °C light and 20 °C dark and were fed on *C. glabra*. Larvae were reared through metamorphosis to the adult life stage. Twenty-four hours after emerging, individuals were sexed and males were put into their assigned treatment group (see below). Butterflies assigned to our minimally processed treatment (freeze-kill, extract) were used as current specimens for comparison to historic specimens as well as to compare to all other treatment groups in the preparation experiment.

Wild *Euphydryas anicia* butterflies were netted from Crescent Meadows in Eldorado Springs, CO (Boulder County) during July 2022 and immediately freeze-killed by placing them into a freezer for 24 h. These butterflies then underwent extraction for determination of iridoid glycoside content and were used in comparison to historic *E. anicia* specimens. Although the host plants of these individuals are unknown, this field location is known to have four catalpol-containing host plants: *Penstemon glaber* var. *alpinus* (Plantaginaceae) and *P. virgatus* (Plantaginaceae), *Plantago lanceolata* (Plantaginaceae) and *Castilleja integra* (Orobanchaceae) (L’Empereur and Stermitz 1990; Kelly and Bowers 2016, 2018).

### Preparation Treatments

Eight preparation treatments, with a sample size of six individuals for each treatment, were used to represent typical preparation techniques for museum specimen butterflies, including a combination or series of the following preparation techniques: use of a killing jar or freeze killing, air-drying or oven-drying after killing, relaxing after drying, and spreading. The eight treatments (*n* = 6 individuals per treatment) were: freeze killing (FE), freeze killing and airdrying (FAE), freeze killing and oven drying (FOE), freeze killing, relaxing jar, and spreading (FRSE), freeze killing and spreading (FSE), killing jar and airdrying (KAE), killing jar, relaxing jar, and spreading (KRSE) and killing jar and spreading (KSE). Time spent in each step was standardized among treatments (Table 1; Fig. S2). Killing jars were saturated with ethyl-acetate to produce a toxic atmosphere for lethal fumigation. Freeze-killing consisted of placing live specimens in a freezer at ~−17 °C for 24 h. Air dried samples were left at ambient room temperature (~ 22 °C) for 4 days (96 h) after death and oven dried samples were placed in a drying oven (equipped with Drierite^®^ desiccant with indicator) at 50 °C for 4 days (96 h) after death. Relaxed samples were placed in a glass jar “relaxer” equipped with soaked sand containing equal parts 70% isopropyl alcohol and deionized water. Sand was soaked until moist to the touch, but not dripping when squeezed. The relaxing jar maintained these conditions throughout the course of the experiment and the sand was rehydrated as needed. Spread samples were pinned with a size 0 or 1 insect pin and pinned to an adjustable wooden spreading board for 4 days (96 h). Small strips (~ 1cmX8cm) of white computer paper were pinned adjacent to the specimen to help stabilize and spread wings. Insect pins were removed from spread specimens and rinsed off into the extraction tube with methanol at the time of extraction. Samples from different treatments spent the allotted time in any one step and immediately were placed in the next step of their treatment.

### Host Plant Samples

Since host plant chemistry is known to influence *Euphydryas* sequestration (Stermitz et al. 1986; Gardner and Stermitz 1988; Mead et al. 1993; Bowers and Williams 1995), we randomly sampled *Chelone glabra* host plants 12 times throughout the course of the *E. phaeton* post-diapause larval rearing for IG quantification. Random samples were taken by selecting ~ 3–5 whole leaves randomly out of a larger bag containing abundant harvested host plant.

### Iridoid Glycoside Extraction

Iridoid glycoside extraction and gas chromatography analyses were completed using previously described methods (Bowers and Stamp 1997). Samples were dried in an oven at ~ 50 °C for 72 h and ground to a course powder, then a ~ 50 mg sample was selected for extraction. Whole butterflies or plant samples were ground with sand and 5mL of methanol using a glass rod. After grinding, test tubes were capped, mixed using a vortexer, and placed on a lab bench at room temperature for 24–48 h. After this initial extraction, samples were filtered to remove sand and sample remnants, and the methanol was evaporated. Then, we added 1mL of 0.500 mg/mL phenyl-β-D-glucopyranoside (PBG) as an internal standard and 3mL of distilled water. We partitioned samples with ether by adding 3mL of ethyl ether, vortexing each sample, then separating the water and ether layers by centrifuging for 4 min. The ether layer, containing lipophilic substances, was removed and discarded; this process was repeated 3 times, the remaining water was evaporated, and samples were resuspended in 1mL methanol and left overnight. A 100µL aliquot was removed from each sample, the methanol evaporated, and the sample derivatized using 100µL Tri-Sil Z (Sigma Aldrich Chemical Company). To quantify the IGs in the samples, we used an Agilent 7890 A gas chromatograph equipped with an FID detector and DB-1 column (30 m, 0.320 mm, 0.25μm particle size). We used an initial temperature of 200 °C held for 1 min, then a 3 min increase to 260 °C which was held for 8 min, followed by a 3 min increase to a final temperature of 320 °C which was held for 10 min.

### Statistical Analysis

All analyses were conducted in R version 2022.07.2 (R Core Team 2022). Percent dry weight of the total IGs were logit transformed in for statistical analyses for both experiments since percent quantities have bounded proportions (between 0 and 100%), however actual concentrations are displayed in figures for interpretability.

*Question 1*: To investigate the effect of specimen age on the ability to detect the presence of iridoid glycosides in preserved museum specimens, we conducted separate linear regression models for each species (*Y* = *β*0​+*β*1​*X*_*1*_​+*ε*; where *X*_*1*_ = year): one nested model on just historic butterflies and another including both historic and current (2023) specimens. We ran these models to investigate both smaller scale differences in chemical sequestration among only the historic specimens as well as overall differences when comparing these historic specimens to current specimens.

*Question 2*: To investigate how preparation technique might impact percent total dry weight IGs we conducted a Kruskal-Wallis one-way analysis of variance with treatment type as a fixed effect.

## Results

**Fig. 1:**
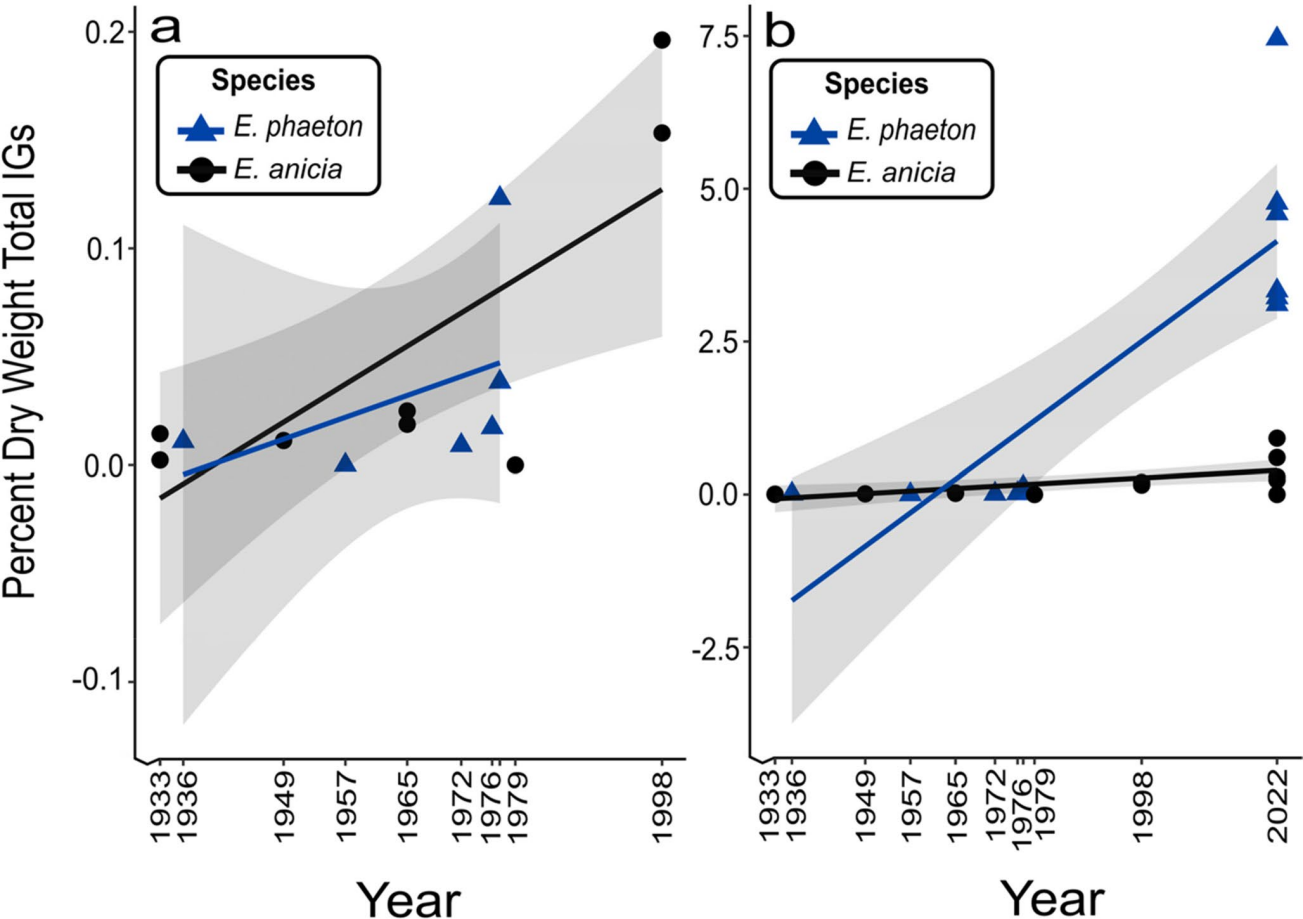
Iridoid glycoside content (percent dry weight total IGs, calculated as (mg IG/mg dry weight of specimen)*100) of **a** historic only butterflies and **b** historic and current butterflies. Linear regressions were run on species separately for each dataset and 95% confidence intervals are shown in grey

*Question 1*: In comparing iridoid glycoside contents of museum specimens, we found that when historic (1933–1998) and current (2022) specimens are pooled there is a significant effect of time on amount of total percent dry weight IGs detected in specimens for both *E. phaeton* (y = −34.17x + 0.016; R^2^ = 0.81; F(1,10) = 41.84; *P* < 0.001) and *E. anicia* (y = −74.13x + 0.036; R^2^ = 0.53; F(1,13) = 14.43; *P* = 0.002) specimens (Fig. 1b). However, when we ran the analyses on only historic specimens (omitting current specimens) we did not see the same pattern for both species. There was a significant effect of time on historic only *E. anicia* (y = − 53.46x + 0.03; R^2^ = 0.61; F(1,7) = 11.13; *P* = 0.013), but this was not consistent for historic only *E. phaeton* (y = − 43.23x + 0.02; R^2^ = 0.26; F(1,4) = 1.396; *P* = 0.3029) (Fig. 1a). Mean ± standard error of total percent dry weight IGs for historic butterflies was 0.047 ± 0.025 for *E. anicia* and 0.033 ± 0.019 for *E. phaeton*. Mean ± standard error of total percent dry weight IGs for historic and current butterflies was 0.440 ± 0.134 for *E. anicia* and 4.412 ± 0.676 for *E. phaeton*.

**Fig. 2:**
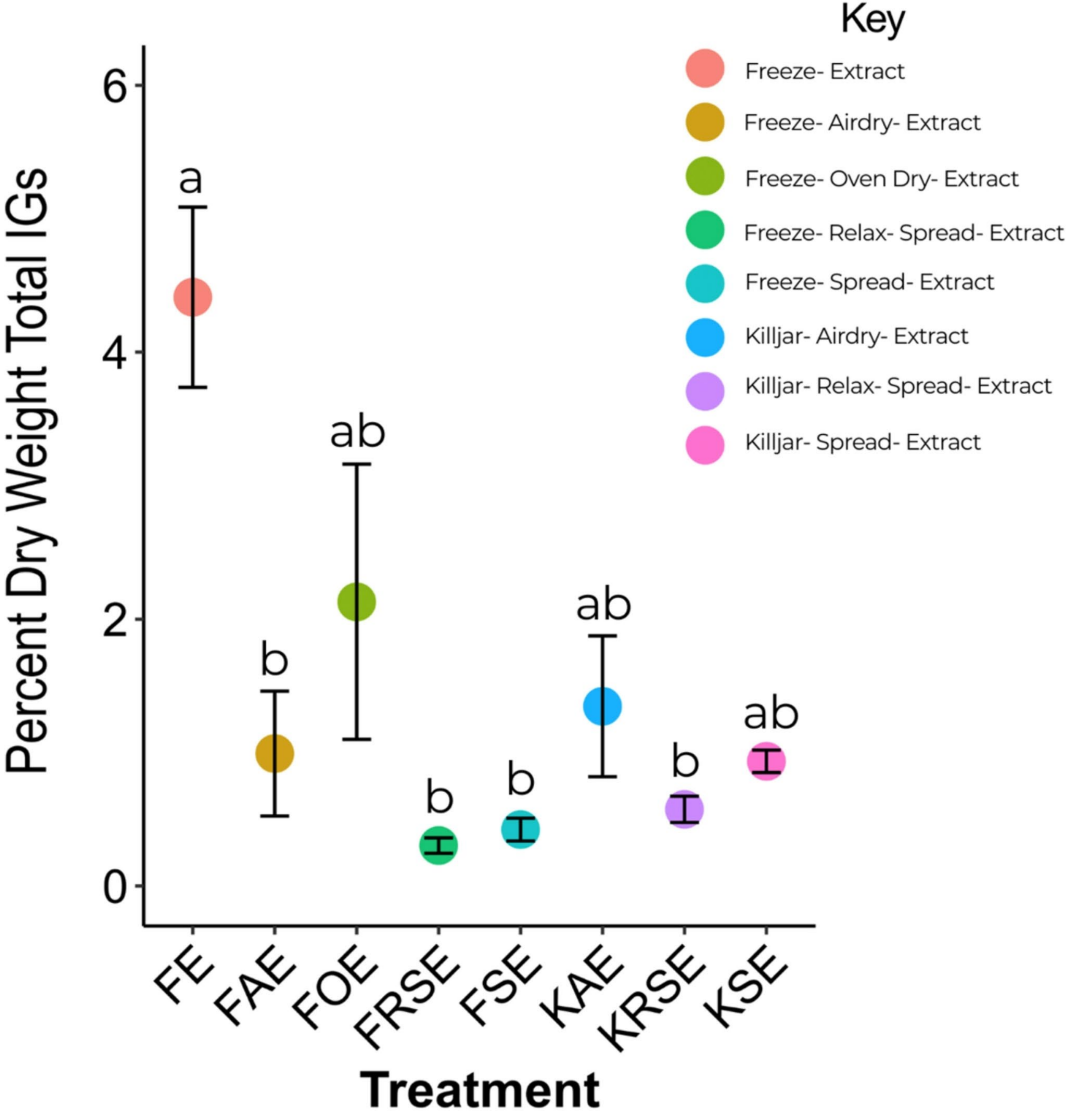
Iridoid glycoside content (percent dry weight total IGs, calculated as (mg IG/mg dry weight of specimen)*100) of butterflies separated by preparation treatment. Treatment group means are expressed by colored data points and SE are shown in black. Treatment components are explained in key. Significant differences resulting from post-hoc Dunn’s Test are shown with different letters over treatments. See Table S3 for significant pairwise differences

*Question 2*: *Do the preparation techniques used in specimen preparation impact iridoid glycoside content?* We first tested for the normality of residuals within treatment groups and found that 3 of the 8 groups were not normally distributed and Levene tests indicated that the data violated the assumption of ANOVA with unequal variances (see Table S2 for assumption tests). Therefore, we ran a non-parametric Kruskal-Wallis one-way analysis of variance and found that treatment had a significant effect on total percent dry weight IGs detected in the butterflies [X^2^ = 24.626, df = 7, *P* < 0.001] and a post-hoc Dunn-test (Bonferroni correction) showed butterflies in the minimally processed group (FE) had significantly higher IG contents than four of the other treatments: FAE, FRSE, FSE, KRSE (*P* < 0.05) (Fig. 2, Table S3), but was not significantly different from the other treatments (FOE, KAE, and KSE). Mean ± standard error of total percent dry weight IGs for the minimally processed treatment group (FE) was 4.41 ± 0.676, which was 3–14 times greater than the other treatments. Treatment had a marginally significant effect on proportion of total iridoid glycosides that was catalpol sequestered by butterflies [X^2^ = 15.9, df = 7, *P* = 0.03] (see Fig. S4 and Table S4 for mean proportion of the total IGs that was catalpol for treatment groups).

Plants were collected 12 times for chemical analysis throughout the course of the larval rearing stage for butterflies used in the preparation treatment experiment (Fig. S3). The range of total percent dry weight IGs for plants was 0.95–5.68 with a mean ± standard error of 3.24 ± 0.34.

## Discussion

Our results demonstrated that historic butterfly museum specimens can be used for detecting sequestered chemical defenses, as we detected sequestered iridoid glycosides in historic museum specimens up to 90 years old. This type of investigation has the potential to provide information about historical plant-insect interactions because sequestered compounds may reflect the make-up of host plants and changes in host plants may result in differences in sequestered compounds. In addition, our results showed that, for preparation techniques that use a form of artificial drying (air-dry or oven-dry), sequestered chemical defenses are minimally affected by type and sequence of preparation technique.

Previous work on the chemical ecology of herbarium specimens has drawn useful conclusions on presence and historic gains and losses of stable compounds such as pyrrolizidine alkaloids (Pelser et al. 2005; Colegate et al. 2014) and furanocoumarins (Tasca et al. 2018; Zangerl and Berenbaum 2005). Only recently has work on museum arthropod specimens been undertaken, primarily investigating cuticular hydrocarbons (Moore et al. 2021; Moris et al. 2021; Soon et al. 2021). Although the iridoid glycosides investigated in the present study, aucubin and catalpol, are considered relatively stable under dry conditions (Gonda et al. 2012), research on factors affecting stability in vivo, such as sequestered compounds, has been limited. However, pH and temperature have been shown to affect stability of other IGs (Ma et al. 2022). Drying samples containing aucubin and catalpol reduces enzyme and microorganism activity, however rehydration (e.g. using a relaxing jar as in this study) can compromise the stability of these compounds through microorganism activation (Kolb 1999). Most of the research on the stability of IGs exists on plants used as herbal supplements (see Kolb 1999; Gonda et al. 2012; Ma et al. 2022), but research on factors affecting stability of these compounds sequestered by insects is limited. Although drying is a common step in entomological specimen preparation, museum specimens are prone to rehydration in some cases (such as relaxing or inadvertent exposure to moist conditions). Our manuscript is the first of our knowledge to tease out the effects of different preparation techniques on the detection of sequestered chemical defenses in entomological specimens and our results show that once dried, sequence and type of preparation techniques minimally affect sequestered defenses.

Although our treatments did not include time as a factor, it is important to note that the treatments which underwent more processing spent more time at ambient room temperature (~ 22 °C) compared to minimally processed samples. It is possible that detection of IGs is impacted by the amount of time spent at these conditions and our results could be reflective of degradation over time at these conditions. However, both aucubin and catalpol have been shown to be relatively stable in rat plasma and cerebral-spinal fluid in relatively short-term storage at room temperature (Wang et al. 2012; Xue et al. 2015). If the detection of IGs is impacted by time spent at room temperature in insect models, there may be a threshold by which specimens begin to degrade, as our minimally processed samples (FE) showed significantly higher amounts of IGs compared to other treatment groups which spent more time in processing. While freeze-drying or may be a better alternative to remove water while avoiding compound degradation, it is seldom used in general insect collection preparation.

Only recently has work using insect museum specimens for chemical ecology been expanded to both identify and quantify chemical compounds, such as lucibufagins in fireflies (Lampryidae) (Berger et al. 2021). We attempted not only to identify whether IGs were present in our insect samples, but also if they were present in amounts large enough to be quantifiable. Although we detected IGs in historic museum specimens up to 90 years old, quantities were in quite low amounts compared to freshly collected specimens. Our results demonstrate that although iridoid glycosides quantities are low in museum specimens, the presence of these compounds can be reliably determined.

One important consideration in this research is the destructive nature of these analyses: because amounts of compounds were small, whole specimens needed to be extracted. This is likely to be true for many other classes of compounds and may limit the utility of this technique for certain taxa, especially for rare specimens. However, research on other compounds using non-destructive sampling methods (such as soaking specimens in methanol) has proven effective (Berger at al. 2021). Previous work has investigated sequestered cycasin (MAM-glycosides) in *Cycas* feeding butterflies using only abdomens from museum specimens and similar extraction techniques as used in the present manuscript (Nash et al. 1992). However, the authors conclude that some suspected sequestering species did not contain detectable MAM-glycoside quantities, most likely due to the advanced age of the specimens. It’s also important to note that other methodology, such as mass-spectrometry, may provide higher resolution than our method of gas chromatography coupled with flame ionization detection and may allow for microsampling from museum specimens.

Our treatments contained relatively low sample sizes for the scope of treatment types (8 treatment types; *n* = 6 for each treatment), which potentially increased type II error. Since we tried to standardize our samples by only using males, we were limited by the number of emerging males available from the lab colony, which consisted of ~ 100 successfully eclosed adults in total. This significantly limited our sample sizes and ability to omit outliers, which may have contributed to the lack of homogeneity of variances between groups. Future work should include non-destructive sampling methods with large sample sizes for treatments and analytical techniques with greater sensitivity.

Although further research on the effects of preparation technique on sequestered defenses is warranted, the present manuscript expands the utility of historic museum insect specimens into the field of chemical ecology. Presence of these compounds in conjunction with associated specimen data, such as date and locality, can inform ecologists about historic conditions, such as potential host-plant species, previous plant communities, associated members of other trophic levels, and potential abiotic conditions. Natural history museum collections hold a wealth of information concerning historic landscapes and ecosystems; expanding this knowledge to include chemical ecology is feasible and should be utilized. While there may be an optimal method for preserving integrity of chemicals found in specimens, the necessities of collections management will typically limit the use of those methods. Entomologists wishing to preserve the chemical compounds of their specimens should take note of these findings and choose the best preservation techniques for their system and compounds of interest.

## Supplementary Information

Below is the link to the electronic supplementary material.

Supplementary 1(DOCX 662 KB)

Supplementary Material 2(DOCX 661 KB)

## Data Availability

No datasets were generated or analysed during the current study.
