# Supplementary material for: Sequestration and the Extended Museum Specimen: Effects of Time and Preparation Methodology
Source: J Chem Ecol. 2025 Sep 29;51(5):96. doi: 10.1007/s10886-025-01646-7 (PMC12479603; doi:10.1007/s10886-025-01646-7)
Supplement: Supplementary file 1 — Supplementary 1(DOCX 662 KB) [file 10886_2025_1646_MOESM1_ESM.docx]

| Table S1. Historic specimen collection information including species, year, county, and state the specimen was collected in. | | | |
| --- | --- | --- | --- |
| Species | Year collected | County collected | State Collected |
| *E. anicia* | 1933 | Gila | Arizona |
| *E. anicia* | 1933 | Gila | Arizona |
| *E. anicia* | 1933 | Gila | Arizona |
| *E. anicia* | 1949 | Boulder | Colorado |
| *E. anicia* | 1965 | Montrose | Colorado |
| *E. anicia* | 1965 | Montrose | Colorado |
| *E. anicia* | 1979 | Park | Wyoming |
| *E. anicia* | 1998 | Boulder | Colorado |
| *E. anicia* | 1998 | Boulder | Colorado |
| *E. phaeton* | 1936 | Berks | Pennsylvania |
| *E. phaeton* | 1958 | Somerset | New Jersey |
| *E. phaeton* | 1972 | Jackson | Missouri |
| *E. phaeton* | 1976 | Coos | New Hampshire |
| *E. phaeton* | 1977 | Franklin | Massachusetts |
| *E. phaeton* | 1977 | Franklin | Massachusetts |

| **Table S2.** Tests for normality and homoscedasticity | | | |
| --- | --- | --- | --- |
| Dataset | Treatment | Test Statistic | Pr(<F) |
| Full | FE | W = 0.8142 | 0.07857 |
|  | FAE | W = 0.80717 | 0.06812 |
|  | FOE | W = 0.77955 | 0.03817 |
|  | FRSE | W = 0.79022 | 0.04791 |
|  | FSE | W = 0.82592 | 0.09923 |
|  | KAE | W = 0.71206 | 0.008263 |
|  | KRSE | W = 0.94314 | 0.6846 |
|  | KSE | W = 0.90496 | 0.4041 |
|  | Levene Test | F = 4.9264 | 0.0004423 |
|  |  |  |  |

| Table S3. Pairwise comparison output for post-hoc Dunn Test for total percent dry weight IGs.  * marks p<0.05, pairwise Z-scores and associated p-values shown. | | | | | | | | |
| --- | --- | --- | --- | --- | --- | --- | --- | --- |
| Dunn’s Test | | | | | | | | |
|  |  | FE | FAE | FOE | FRSE | FSE | KAE | KRSE |
| FAE |  | 3.051708 |  |  |  |  |  |  |
|  | p | 0.0319* |  |  |  |  |  |  |
| FOE |  | 1.876388 | -1.17532 |  |  |  |  |  |
|  | p | 0.8484 | 1 |  |  |  |  |  |
| FRSE |  | 4.227028 | 1.17532 | 2.35064 |  |  |  |  |
|  | p | 0.0003* | 1 | 0.2624 |  |  |  |  |
| FSE |  | 3.629058 | 0.57735 | 1.75267 | -0.597969 |  |  |  |
|  | p | 0.0040* | 1 | 1 | 1 |  |  |  |
| KAE |  | 1.876388 | -1.17532 | 0 | -2.35064 | -1.75267 |  |  |
|  | p | 0.8484 | 1 | 1 | 1 | 1 |  |  |
| KRSE |  | 3.010469 | -0.041239 | 1.13408 | -1.216559 | -0.618569 | 1.13408 |  |
|  | p | 0.0365* | 1 | 1 | 1 | 1 | 1 |  |
| KSE |  | 1.793909 | -1.257798 | -0.082478 | -2.433118 | -1.835149 | -0.082478 | -1.216559 |
|  | p | 1 | 1 | 1 | 0.2096 | 0.9308 | 1 | 1 |

| Table S4. Pairwise comparison output for post-hoc Dunn Test for proportion of IGs that was catalpol.  * marks p<0.05, pairwise Z-scores and associated p-values shown. | | | | | | | | |
| --- | --- | --- | --- | --- | --- | --- | --- | --- |
| Dunn’s Test | | | | | | | | |
|  |  | FE | FAE | FOE | FRSE | FSE | KAE | KRSE |
| FAE |  | 1.979486 |  |  |  |  |  |  |
|  | p | 0.6687 |  |  |  |  |  |  |
| FOE |  | 1.134080 | -0.845405 |  |  |  |  |  |
|  | p | 1 | 1 |  |  |  |  |  |
| FRSE |  | 3.237385 | 1.257798 | 2.103204 |  |  |  |  |
|  | p | 0.0169* | 1 | 0.4963 |  |  |  |  |
| FSE |  | 1.690811 | -0.288675 | 0.556730 | -1.546473 |  |  |  |
|  | p | 1 | 1 | 1 | 1 |  |  |  |
| KAE |  | 0.206196 | -1.773290 | -0.927884 | -3.031088 | -1.484614 |  |  |
|  | p | 1 | 1 | 1 | 0.0341* | 1 |  |  |
| KRSE |  | 2.268161 | 0.288675 | 1.134080 | -0.969123 | 0.577350 | 2.061965 |  |
|  | p | 0.3265 | 1 | 1 | 1 | 1 | 0.5490 |  |
| KSE |  | 1.360897 | -0.618589 | 0.226816 | -1.876388 | -0.329914 | 1.154700 | -0.907264 |
|  | p | 1 | 1 | 1 | 0.8484 | 1 | 1 | 1 |

**Figure Legends**

**Fig. S1** Experimental design schematic for historic specimens (*note not all dates are included in schematic). Both *E. phaeton* and *E. anicia* Specimens were chosen from University of Colorado’s Museum of Natural History entomology collections from collection dates with multiple individuals. Actual date and collection information for specimens can be found in Table 1.

**Fig. S2** Experimental design schematic for preparation treatment experiment. Symbols represent different components of the preparation technique connected by lines to represent the different treatments. Treatment components are labeled to the left of each treatment type. All specimens began as fresh live specimens and concluded treatment with sequestration analysis.

**Fig. S3** Iridoid glycoside content (percent dry weight total IGs, calculated as (mg IG/mg dry weight of specimen)*100) of *Chelone glbara* at harvest for feeding caterpillars over the course of the experiments. A single plant sample consisted of multiple leaf pieces that were analyzed at each date. Bars show percent dry weight iridoid glycoside content for aucubin and catalpol pooled at each collection date.

**Fig. S4** Proportion catalpol calculated as (mg catalpol/mg total IGs (mg aucubin + mg catalpol)) of butterflies separated by treatment group. Treatment group means are expressed by colored data points and SE are shown in black. Treatment components are explained in key. Significant differences resulting from post-hoc Dunn’s Test are shown with different letters over treatments. See Table S4 for significant pairwise differences.

**Figures**

**
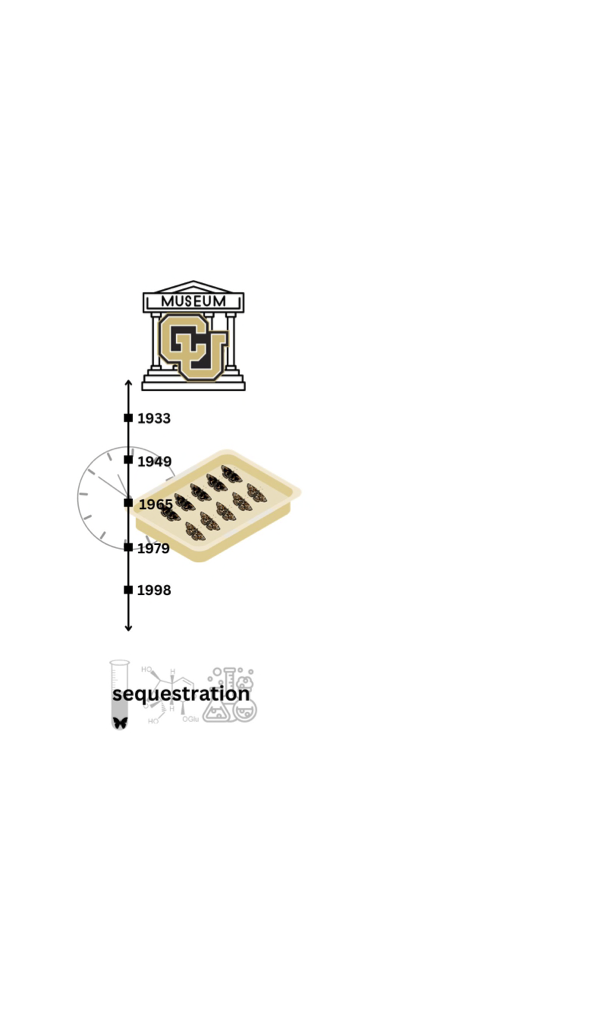
**

**Fig. S1**

**
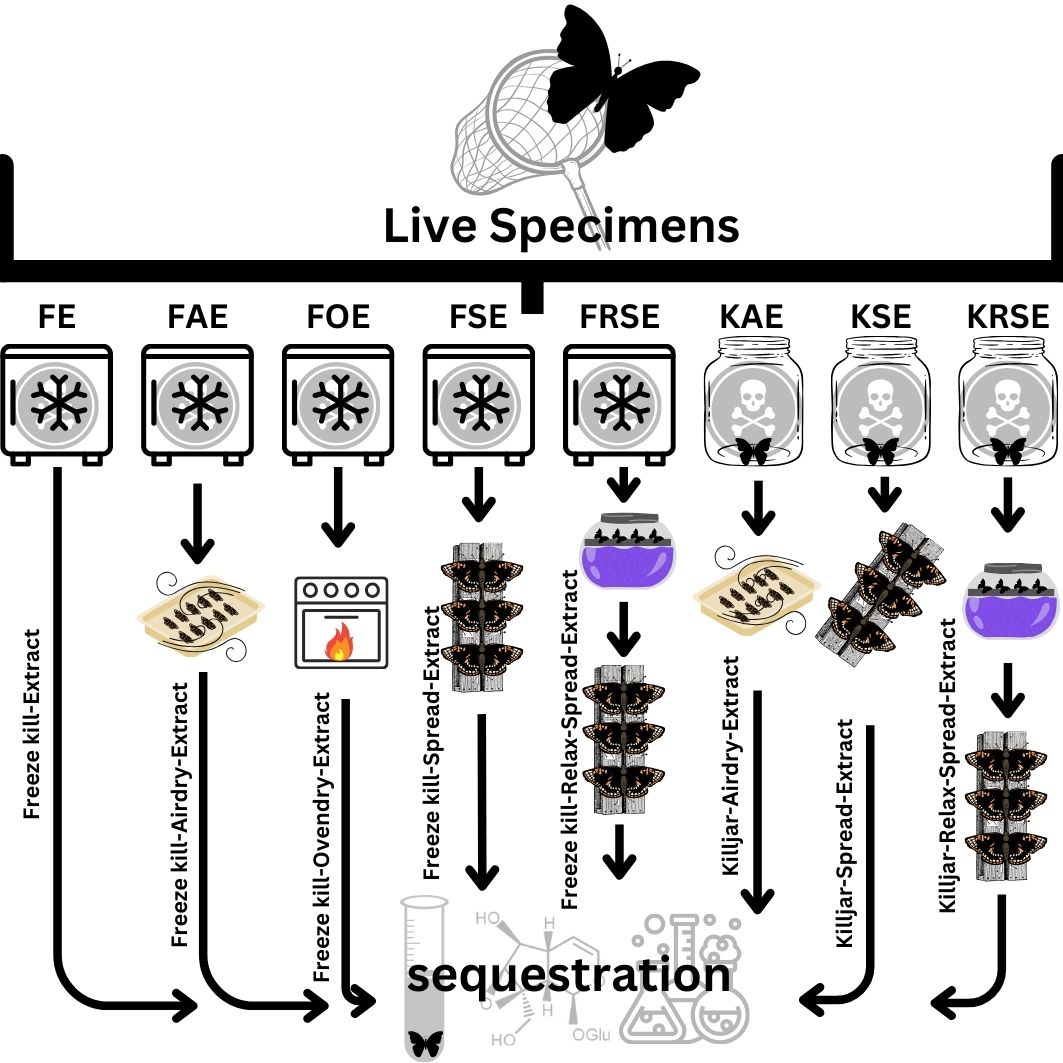
**

**Fig. S2**

**
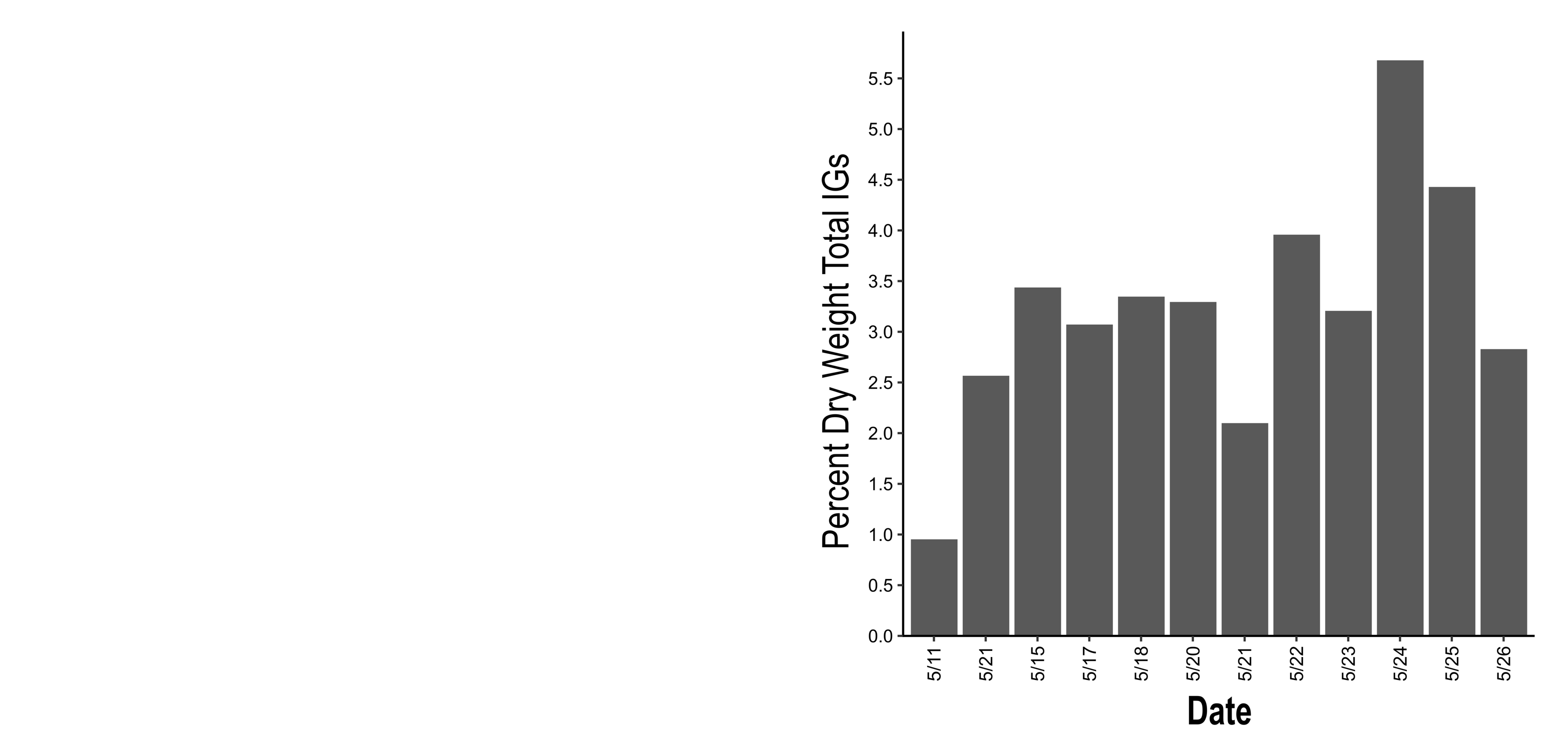
**

**Fig. S3**

**Fig. S4
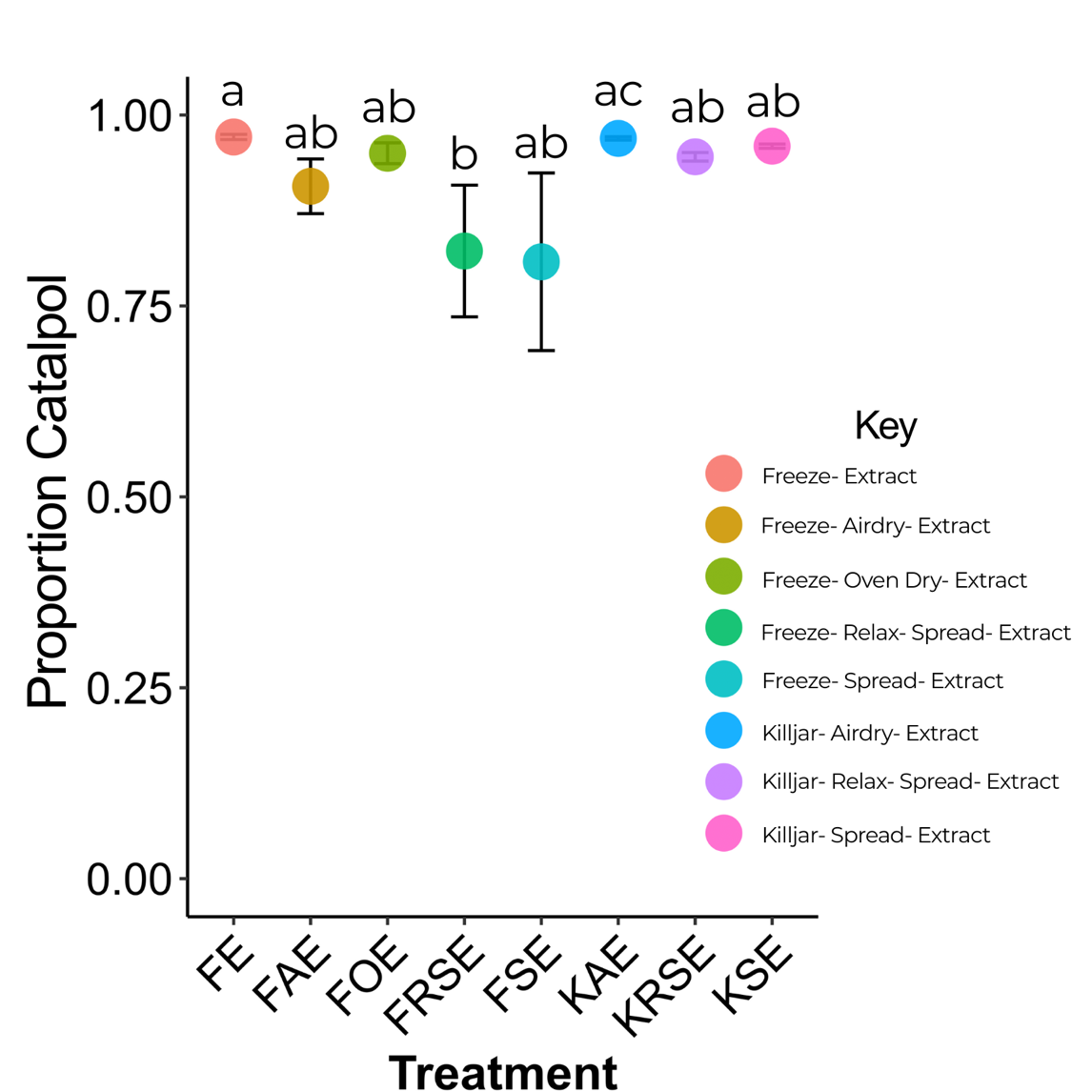
**
